# Medical and Philosophical Intelligence

**Published:** 1819-05

**Authors:** 


### MEDICAL AND PHILOSOPHICAL INTELLIGENCE.

A CASE of *Tetanus* is related in the last Number of the Edinburgh Medical and Surgical Journal, by Dr. DOUGLAS, induced, apparently, from the influence of cold after the patient had been much heated and fatigued by husbandry labour; which terminated favourably under the use of a very large dose of opium (taken from mistake), followed by active purgatives. Bleeding had been once employed; sudorifics, opium, and purgatives, in moderate doses, given; a blister applied to the sternum, the most painful part; and afterwards, with the same measures, æther, tincture of castor, calomel, and the warm bath, without benefit; when, on the fifth day of the disease, the patient took at intervals, in one night, a mixture, intended for an embrocation, composed of two ounces and a half of tincture of opium, two drachms of sulphuric æther, and two drachms of camphorated spirits; the next morning the spasms were perfectly relieved. Active purgatives were then administered: the report of the following day is—"He had three stools during the night, (he also had three during the preceding day,) *dark-coloured and fœtid;* he did not sleep much, but was free from spasms. The sardiasis is still particularly striking when he swallows, which he says gives him pain." Friction with laudanum, &c. over the sternum, which was still very painful, and the use of a solution of sulphate of magnesia internally, were continued; and, in about a week afterwards, he was getting tolerably well.

---

The same Journal contains the history of a case of *Hydrophobia*, by Dr. James Johnson, of Dundee. The patient, a girl nine years of age, was bitten on the cheek by a rabid dog: the wounds were but slight, little more than through the cuticle; the parts were not excised until seventy hours after the accident, when Dr. Johnson first saw the patient. About the third week after the injury she was observed to have disturbed sleep; and in five weeks the characteristic symptom appeared. The case terminated fatally. Mercurial friction was the only measure that was employed. Examination of the body after death was not effected. We shall detail a few of the symptoms, which we consider particularly interesting:—Much pain was felt at the pit of the stomach. The heart palpitated; a cold hand applied to the region of the heart induced a deep sigh, and increased the pain at the stomach. No pain was felt at the throat. The intellectual faculties were not disturbed. "The application of a glyster-pipe to the extremity of the rectum brought on most severe spasms, and the outlet of the bowels was so constricted that it seemed as impracticable to introduce the pipe into the rectum as it would have been to push it through the parietes of the abdomen."

A short time before death she drank wine and water "greedily, and without feeling any uneasiness: she rejoiced, with her mother, at being able to drink, and had herself carried to a neighbouring house, to show that she was much better. An hour afterwards, she began to talk incessantly, and attempted to sing; by which she appeared to exhaust herself. She again became very low, and sunk. The spasms were frequent and severe; they seemed to degenerate into a constant sprawling, unmeaning, motion of the arms and legs, which was terminated by two unsuccessful efforts to vomit; with which she ceased to struggle, and instantly expired, without any noise or motion indicative of pain."

The pulse, during the disease, arose to 140, and was feeble.

The skin was not hot; but a linen sheet was, for the most part, her only covering, (in the month of January.) "Respiration was performed easily, except during the fits."

---

*Cow-Pock in Persia.*—The following account of the cow-pock in Persia is from a letter written by W. Bruce, esq. resident at Bushire, to W. Erskine, esq. of Bombay, March 1813.

"When I was in Bombay, I mentioned to you that the cow-pock was well known in Persia, by the Eliaats, or wandering tribes. Since my return here, I have made very particular enquiries on the subject, amongst several different tribes who visit this place in the winter, to sell the produce of their flocks; such as carpets, rugs, butter, cheese, &c. Their flocks during this time are spread over the low country to graze. Every Eliaat that I have spoken to on this head, of at least six or seven different tribes, has uniformly told me that the people who are employed to milk the cattle caught a disease, which after once having had they were perfectly safe from the small-pox; that this disease was prevalent among the cows, and shewed itself particularly on the teats; but that it was more prevalent among, and more frequently caught from, the sheep. Now this is a circumstance that has never, I believe, before been known, and of the truth of it I have not the smallest doubt, as the persons of whom I enquired could have no interest in telling me a falsehood; and it is not likely that every one whom I spoke to should agree in deceiving, for I have asked at least near forty or fifty persons. To be more sure on the subject, I made most particular enquiries of a very respectable farmer who lives about fourteen miles from this, by name Malalla, and who is under some obligations to me: this man confirmed every thing that the Eliaats had told me, and further said, that the disease was very common all over the country, and that his own sheep often had it. There may be one reason for the Eliaats saying that they caught the infection oftener from the sheep than the cow; which is, that most of the butter, ghee, cheese, &c. is made from sheep's milk, and that the black cattle yield very little, being more used for draught than any thing else. —*Philosophical Magazine.*

---

A society has been established in London, bearing the designation of the "*Hunterian Society.*" It professes the most friendly feeling towards all similar existing institutions, and is founded principally, but not exclusively, for the accommodation and benefit of medical men residing in the eastern parts of the metropolis. Its objects are to concentrate the zeal and experience of a large number of respectable practitioners, whose places of residence are at a distance from professional associations already existing, and to receive and discuss communications on medical, chirurgical, and all other subjects connected with medicine. It aims particularly at the cultivation of a spirit of liberal and friendly intercourse among the members of the profession within the sphere of its influence.

It consists of honorary, corresponding, and ordinary, members; and already the Society is honoured by the names of a considerable number of men of character and talent. The following is a list of the officers and council for the present year:—

*President*—Sir William Blizard, F.R.S.

*Vice-Presidents*—James Hamilton, M.D. John Meyer, M.D. George Vaux, Esq. Lewis Leese, Esq.

*Treasurer*—Benjamin Robinson, M.D.

*Secretaries*—John T. Conquest, M.D. F.L.S. Thos. J. Armiger, Esq.

*Council*—Thomas Addison, M.D. Thomas Bell, Esq. F.L S. Henry James Cholmley, M.D. Thomas Calloway, Esq. William Cooke, Esq. George Edwards, Esq. James Alexander Gordon, M.D. William Kingdon, Esq. Benjamin Pierce, M.D. James Parkinson, Esq. Henry Richard Salmon, Esq. Frederick Tyrell, Esq.

The Hunterian Society holds its meetings every alternate Wednesday evening throughout the year, at No. 10, St. Mary Axe; where communications on any department of medical science, addressed to the secretaries, will be thankfully received.

---

*Observations on Phosphorescence in Terrestrial Animals.*—This should be distinguished from the electricity sometimes spontaneously developed in individuals who have used much exercise, from whose dry hair sparks of light will be evolved after friction. This phenomenon, as it relates to cats, especially in winter, is almost universally known. Many analogous observations respecting it was made by the ancients; for to this should be referred that gleam of the hair of Achilles when in violent anger, described by Homer; and that lambent flame which, as Virgil says, seemed to encircle the head of Ascagnius:—"*Lambere flamma comas et circùm tempora pasci.*" Grooms talk of the sprites that sometimes appear on the manes of horses, especially when they are curried. Some authors state that they have remarked signs of electricity also on the plumage of some parroquets, &c.

We should, perhaps, attribute to this development of electricity the instances of spontaneous combustion of several fat individuals, habituated to the use of spirituous liquors; such as women exhaling hydrogenated and inflammable gases, &c.

There is another mode of lucidity, which appears to depend more immediately on nervous action: it is that evinced in the eyes of cats, wolves, owls, and other nocturnal animals. Their pupil dilates considerably in obscurity, and the retina, the appearance of which is shining, sends forth manifest light. We have had occasion to observe this effect in a fox mortally wounded, who, on his being seized in the obscure retreat to which he had fled, opened his pupils to an extraordinary extent, and developed flaming eyes. It appears that the same effect is produced in those glances during anger which are commonly termed fiery: it is thus with the eyes of a cat plunged into water; the terror dilates the pupil, and the retina gives forth luminous rays like a mirror opposed to the sun. We shall not adduce the observations of some ancient philosophers,—such as Plato, Epicurus, &c.—who supposed that light was darted by the eye, and acted on other individuals, in love, envy, anger; and that persons might thus be fascinated, as the look of the dog arrests the partridge.

"*Nescio quis teneros oculus mihi fascinat agnos.*"

Amongst reptiles, it is pretended that crocodiles and nocturnal lizards dart sparkling glances during the night. Perhaps we might attribute to the same origin the fear caused by serpents, whence the ancients imagined the basilisk killed by his look alone; and it has been pretended that the sudden sight of a wolf caused a loss of voice.

The true animal *phosphorescence* is particularly observed in the class of insects, especially in the coleopteres. In this order are distinguished the genera *elater*, *lampyris*, and *paussus*. The *elater noctilucus*, the *cucujo* of the Americans, or the *fire-fly*, has the faculty, according to PATRICK BROWNE (*Hist. of Jamaica*), to suspend, or to develop, light at will, just as it can be done with a dark lantern. The phosphorescent organs are situated in the corselet on each side, and are retracted during fear. The corselet contains a good deal of luminous matter of a yellowish colour, which is semi-transparent and gelatinous. This insect, which hides itself during the day, flies about during the night, and throws itself into the flame of burning lamps, &c. Its light is so vivid, that a person can read with the aid of eight or ten of these flies as well as with a candle, or travel abroad at night by the glow they diffuse. The Indians take them about in journeys instead of lanterns; and the women ornament their heads with them, as with brilliant stars.

Two other varieties of the phosphorescent elater are known in South America and the Antilles: the *elater phosphoreus* of GEER, and the *elater ignitus* of FABRICIUS, (*Journal de Pharmacie*, Jan. 1819.)

(*To be continued.*)

---

WE have received a letter, signed "TYRONES," from some young students in medicine, who are now in the latter term of their pupillage to different practitioners in a town in Devonshire, who appear to feel a zeal for the acquisition of professional knowledge which hardly ever fails to lead to what is alone sufficient to compensate for a life devoted to arduous, though gratifying, labours. "They have formed a society, of which a meeting is held twice a week, from six to eight each evening; at each of which a lecture (composed of selections from the best writers on materia medica, pharmacy, rudiments of anatomy and physiology, practice of physic, &c. to which subjects they for the present confine themselves,) is read, and suitable demonstrations given by the member whose turn it may be; which being finished, the members criticise on the several errors committed by the lecturer, and discuss, as far as capacity will allow, on the subject and substance of the lecture. This done, each member is required to put forward at least twelve questions on some of the before-mentioned subjects; each of which questions, if not answered by one or more of his fellow-members, is solved by himself. These proceedings occupy, at least, the allotted two hours."

Their intention, in transmitting this letter, and ours in inserting it in our Journal, is to request the advice of any of our readers respecting means which they consider adapted to the improvement of the mode of conduct they have adopted. We hope the adducing so laudable an example will incite other of our young readers in the same situation, and who may have similar opportunities, to adopt the same measures. Whenever there are twelve pupils, who have passed the middle of the term of their apprenticeship, in any town in England, such a society should be formed: let them remember that—"Nihil autem est amabilius quam morum similitudo bonorum; in quibus enim eadem studia sunt, eademque voluntates; in his fit, ut aeque quisque alter delectetur, ac seipso."

### MONTHLY CATALOGUE OF MEDICAL BOOKS.

### REPORT OF DISEASES.

THE numerous variations which have taken place in the state of the atmosphere during the last month, have given rise to nearly equally various characters in the form of the most prevalent diseases; but, as is usual at this season, the far greater proportion of them has been essentially of an inflammatory character: we say *essentially*, because, as there are no, or at least but very few, diseases that are not accompanied in some stage or other with increased sensibility, either organic or animal (using these terms according to the doctrine of BICHAT), so are there hardly any disorders that are not at some period attended with more or less of inflammatory action.

Typhous fever, whether apparently arising from contagion or other causes, has evinced during life more evident signs of inflammation of the mucous membranes of the gastric organs than it did during some months previously. The extraordinary effects which we have witnessed from the application of leeches to the upper region of the abdomen, has led us to consider this as preferable to venesection in the greater number of cases, even in the first stage of the disease: the counter-irritation caused by the bites of the leeches may, probably, contribute to the good effects they produce. That there exists great and particular sympathy,—or synenergy, as Professor GRIMAUD, of Montpellier, used to term it, with more propriety, in his lectures,—between the intestines and abdomen, is shown by numerous physiological and pathological phenomena. The use of leeches cannot, however, become general for this purpose in England, as it is necessary that from twenty to forty should be applied at once: perhaps, cupping would be nearly equally beneficial.

Various forms of thoracic inflammation continue to be prevalent. Hæmorrhages, from various parts, are of frequent occurrence: these, as well as some other analogous affections, would perhaps be avoided by the old custom of taking a course of purgative medicine at this season. We do not believe that they act as alterants, according to popular notions, but they cause a constant determination to a part which attracts, (to use a metaphorical expression,) and carries off, without permanent injury to the system, the undue excitement to which persons of a plethoric habit are so very liable at this period of the year. This remark may be illustrated by some of the effects of mercury. The French practitioners consider that they can cause the influence of this medicine to be chiefly exerted on any part they desire, merely by the use of some preparatory measures, —as the maintaining a certain degree of topical irritation previously to its use: they would, indeed, think themselves great bunglers in the practice of the medical art if they could not, when they pleased, prevent salivation, and yet obtain all the desired effects of the remedy, in the greater proportion of instances. On looking into our manuscript transcript of the lectures of Mr. PEARSON, we find a somewhat similar observation.

Chronic diseases should be cautiously watched at this season, as they are particularly disposed to pass rapidly into a state of increased or inflammatory action.

We have recently observed a curious instance of the effects of friction with the tartar-emetic ointment. On advising the use of it for a female with chronic inflammation of the uterus, we found that it invariably excited vomiting. This, as far as one case will permit, with the support of other analogous phenomena,—as inflammation of the stomach, expressly, from the external application of arsenic; the purging caused by that of jalap, scammony, &c.—will shew that tartar-emetic may be used externally with other views besides that of inducing counter-irritation.

### METEOROLOGICAL JOURNAL,

### By Messrs. WILLIAM HARRIS and Co. 50, Holborn, London.

### *From the 20th March to the 19th April, 1819, inclusive.*

| Day of Month. | Moon. | Rain gauge | Therm. | | | Barom. | | De Luc's Hygrom. Dry | Damp | | Wind. | | Atmospheric Variation. | | |
|---|---|---|---|---|---|---|---|---|---|---|---|---|---|---|---|
| Mar. | | | | | | | | | | | | | | | |
| 20 | | | 45 | 50 | 44 | 29·48 | 29·72 | | 9 | 10 | NNW | NNW | Rain | Clo. | |
| 21 | | ,05 | 46 | 49 | 42 | 29·80 | 29·85 | | 10 | 11 | N | SE | Fine | | |
| 22 | | | 47 | 51 | 44 | 29 84 | 29·81 | | 11 | 10 | W va. | W va. | Fine | | Clo. |
| 23 | | | 47 | 50 | 45 | 29·77 | 29·69 | | 8 | 11 | SW | SSE | Clo. | | |
| 24 | | | 48 | 55 | 47 | 29·59 | 29·71 | | 12 | 11 | WSW | SW | Rain | Fine | |
| 25 | ● | ,05 | 50 | 54 | 41 | 29·70 | 29·74 | | 11 | 11 | W | W va | Rain | Shory | Fine |
| 26 | | ,04 | 45 | 50 | 45 | 29·85 | 29·99 | | 11 | 9 | W | W | Fine | | Shory |
| 27 | | ,03 | 49 | 51 | 47 | 29·91 | 29·86 | | 8 | 7 | SW va | W va. | Fine | Rain | Clo. |
| 28 | | ,24 | 51 | 57 | 49 | 29·85 | 29.81 | | 9 | 10 | SW | SW | Rain | Clo. | |
| 29 | | | 51 | 53 | 46 | 29·75 | 29·87 | | 10 | 10 | WSW | W va. | Fine | Rain | Fine |
| 30 | | ,03 | 50 | 55 | 47 | 29·75 | 30·04 | | 11 | 11 | SW | W va. | Rain | | |
| 31 | | ,12 | 54 | 58 | 47 | 30·10 | 30·14 | | 13 | 11 | WNW | WSW | Clo. | | |
| Apr. | | | | | | | | | | | | | | | |
| 1 | | | 54 | 59 | 48 | 30·16 | 30·16 | | 10 | 12 | W | WNW | Fine | | |
| 2 | ○ | | 55 | 62 | 47 | 30·15 | 30·12 | | 11 | 10 | NW | SE | Fine | | |
| 3 | | | 53 | 63 | 47 | 30·04 | 30·00 | | 10 | 9 | W | SE | Fine | | |
| 4 | | | 52 | 57 | 45 | 30·05 | 30·08 | | 9 | 10 | SE | SE | Fine | | |
| 5 | | | 51 | 56 | 41 | 30·09 | 30·05 | | 8 | 8 | NE | SE | Fine | | |
| 6 | | | 45 | 53 | 46 | 29·93 | 29·74 | | 7 | 8 | SE | SE va. | Fine | | |
| 7 | | | 50 | 61 | 50 | 29·71 | 29·78 | | 8 | 9 | SSE | SE | Fine | | Shory |
| 8 | | | 54 | 54 | 45 | 29·86 | 29·96 | | 11 | 10 | S | N | Fog | Rain | Fine |
| 9 | | | 50 | 58 | 47 | 30·03 | 30·07 | | 10 | 10 | N | N | Fine | | |
| 10 | ○ | ,32 | 51 | 57 | 47 | 30·05 | 29·78 | | 7 | 6 | N | W | Fine | | |
| 11 | | | 51 | 56 | 44 | 29·47 | 29·41 | | 7 | 8 | WSW | W | Fine | | |
| 12 | | | 45 | 52 | 49 | 29·39 | 29·32 | | 10 | 12 | W | NW | Clo. | Rain | |
| 13 | | ,26 | 52 | 55 | 45 | 29·27 | 29·51 | | 13 | 10 | ESE | SSW | Fog | Rain | Fine |
| 14 | | ,19 | 47 | 53 | 45 | 29·50 | 29·44 | | 8 | 8 | SW | SW | Clo. | Fine | Rain |
| 15 | | ,23 | 48 | 54 | 47 | 29·46 | 29·41 | | 7 | 7 | WSW | SE | Shory | Fine | Clo. |
| 16 | | ,09 | 49 | 55 | 44 | 29·37 | 29·33 | | 8 | 10 | SW | SW | Shory | | |
| 17 | ◑ | ,15 | 48 | 54 | 49 | 29·42 | 29·52 | | 7 | 9 | SW | SW | Shory | Stormy | Shory |
| 18 | | ,17 | 51 | 58 | 48 | 29·55 | 29·50 | | 10 | 11 | WSW | WSW | Fine | Shory | Clo. |
| 19 | | ,31 | 50 | 55 | 49 | 29·65 | 29 69 | | 10 | 11 | SW | W | Fine | Shory | Rain |

The quantity of rain fallen in the month of March
is 1 inch and 15-100ths.

