# Corrigenda

**Published:** 1819-01

**Authors:** 


					449, , for Dr. Dovgluss read Dr. Hall.
J. and C. Adlard, Printer?,
33, Bartholomew Clos*.

				

